# Protein-Based Nanoparticle Preparation via Nanoprecipitation Method

**DOI:** 10.3390/ma11030394

**Published:** 2018-03-07

**Authors:** Mohamad Tarhini, Ihsane Benlyamani, Selim Hamdani, Géraldine Agusti, Hatem Fessi, Hélène Greige-Gerges, Abderrazzak Bentaher, Abdelhamid Elaissari

**Affiliations:** 1 Univ Lyon, Université Claude Bernard Lyon-1, CNRS, LAGEP UMR 5007, 43 boulevard du 11 Novembre 1918, F-69100 Villeurbanne, France; mohamad.tarhini@etu.univ-lyon1.fr (M.T.); ihsanebell@gmail.com (I.B.); hamid2002@live.fr (S.H.); geraldine.agusti@univ-lyon1.fr (G.A.); hatem.fessi@univ-lyon1.fr (H.F.); 2 Faculty of Sciences, Lebanese University, B.P. 90656 Jdaidet El-Matn, Lebanon; hgreige@ul.edu.lb; 3 Inflammation and Immunity of the Respiratory Epithelium-EA 7426, Faculté de Médecine Lyon Sud, 69495 Pierre Benite, France; azzak.bentaher@inserm.fr

**Keywords:** bovine serum albumin, colloids, nanoparticles, nanoprecipitation, size

## Abstract

Nanoparticles are nowadays largely investigated in the field of drug delivery. Among nanoparticles, protein-based particles are of paramount importance since they are natural, biodegradable, biocompatible, and nontoxic. There are several methods to prepare proteins containing nanoparticles, but only a few studies have been dedicated to the preparation of protein- based nanoparticles. Then, the aim of this work was to report on the preparation of bovine serum albumin (BSA)-based nanoparticles using a well-defined nanoprecipitation process. Special attention has been dedicated to a systematic study in order to understand separately the effect of each operating parameter of the method (such as protein concentration, solvent/non-solvent volume ratio, non-solvent injection rate, ionic strength of the buffer solution, pH, and cross-linking) on the colloidal properties of the obtained nanoparticles. In addition, the mixing processes (batch or drop-wise) were also investigated. Using a well-defined formulation, submicron protein-based nanoparticles have been obtained. All prepared particles have been characterized in terms of size, size distribution, morphology, and electrokinetic properties. In addition, the stability of nanoparticles was investigated using Ultraviolet (UV) scan and electrophoresis, and the optimal conditions for preparing BSA nanoparticles by the nanoprecipitation method were concluded.

## 1. Introduction

Nanoparticles are generally submicron nano-sized materials that can be obtained from a variety of synthetic and natural materials. They include carbon nanotubes, polymers, lipid-based and peptide-based nanoparticles, etc. These particles may have optical, electronic, and catalytic properties [1]. Regarding polymer particles dedicated for in vivo applications, protein-based nanoparticles show various advantages; they are biodegradable, nontoxic, have low antigenicity, and provide large possibilities for surface modification and covalent binding of drugs. In addition, various kinds of proteins are easy to obtain from numerous sources [1,2].

From the wide variety of proteins generally used to prepare nanoparticles, albumin has been extensively used due to its availability, innocuous degradation metabolites, water solubility, and availability in pure form. All these advantages make it perfect for preparation of nanoparticle dispersions [3]. Moreover, the presence of multiple binding sites in its structure improves its binding capacity to various active molecules [4]. There are different types of albumin according to its origin. The well-studied albumins are human serum albumin (HSA), bovine serum albumin (BSA), and ovalbumin. Recently, special attention has been dedicated to the nanoparticle formulation from albumin macromolecules. In fact, some formulations such as Abraxane^®^ (paclitaxel loaded albumin nanoparticles) are nowadays commercially available [3]. Moreover, albumin’s primary structure has a high content of charged amino acid residues, which could lead to attractive electrostatic interaction with positively- or negatively-charged active molecules on protein-based nanoparticles without the addition of any intermediate molecules [5]. The majority of reported studies have been focused on BSA since it has good acceptance in the pharmaceutical industry and is used as a carrier system or just as a protein model in numerous fundamental studies [6,7,8,9,10,11].

BSA-based nanoparticles can be prepared by different methods that can be divided into two main categories: emulsification and precipitation. Methods such as coacervation, water-in-oil emulsion, and desolvation are widely used in previous works [12]. Table 1 represents different BSA formulations prepared by different methods to encapsulate various active molecules.

Nanoprecipitation is based on the reduction of the quality of the solvent in which the main constituent of nanoparticles is dissolved. Such variation in solvent quality can be achieved by altering the pH, salt concentration, solubility conditions, or the addition of a non-solvent [13]. Depending on the approach used to reduce the solvent quality, nanoprecipitation can be classified into different submethods such as: non-solvent precipitation, desolvation, coacervation, salting out, and albumin bound technology.

**Figure 1:**
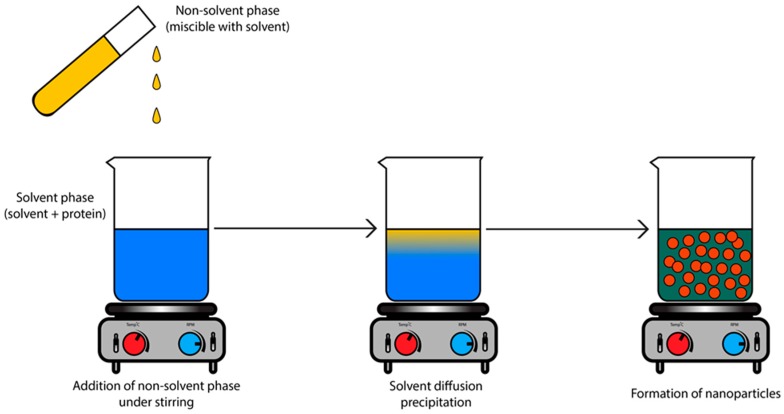
Protein-based nanoparticles prepared by nanoprecipitation method leading to submicron particles. The addition of the non-solvent phase leads to a state of supersaturation which allows the beginning of the nanoprecipitation process and the formation of nanoparticles.

This work is based on the non-solvent precipitation method. In brief, protein is dissolved in an aqueous solvent while ethanol is used as a poor solvent for the protein with good water miscibility (Figure 1). By mixing both phases, supersaturation of the protein occurs, followed by the formation of protein nuclei. The free protein units will then condense around the nuclei, creating protein nanoparticles. This technique is known to produce small-sized particles with unimodal distribution. In addition, this method does not require the use of toxic compounds such as glutaraldehyde, usually used as a crosslinker, and it is famous for the encapsulation of water-soluble drugs [14].

Moreover, some studies reported successful encapsulation of hydrophobic drugs into polymer nanoparticles using this technique [15,16]. The non-solvent-based precipitation process includes three steps: generation of supersaturation, nucleation, and growth. Each step is affected by one or more of the experimental parameters, and the physicochemical properties of nanoparticles are affected by the rate and the behavior of the formulation in each precipitation step. Therefore, the experimental parameters could affect the physicochemical properties of nanoparticles [17,18].

Various parameters affecting the nanoprecipitation process have been investigated in the literature [30]. Tyrphostin-loaded polylactic acid nanoparticles were prepared by the nanoprecipitation method [31] and it was found that by increasing the polymer amount from 100 (5 mg/mL) to 300 mg (15 mg/mL), the hydrodynamic particle size increases from 70 to 140 nm. Surprisingly, for a polymer amount lower than 150 mg, the colloidal stability of the formulated dispersion was affected [31].

In another study, polylactic acid nanoparticles were also investigated [32]. It was found that by increasing the polymer concentration from 5 to 66.6 mg/mL, the size of nanoparticles increased gradually from 200 to 300 nm with maintained unimodal size distribution. On the other hand, the decrease of the solvent/non-solvent volume ratio led to a reduction in the particle size [32]. However, for polycaprolactone nanoparticles, the decrease of the solvent/non-solvent volume ratio led to an increase in the particle size [33]. This indicates that the effect of parameter modification can differ depending on the nature of polymers. Moreover, the effect of agitation rate on the polylactic acid nanoparticles was also studied [34]. The increase of agitation speed caused a reduction in particle size since faster agitation can trigger a faster diffusion rate which leads to smaller particles [34].

Then, this work aims to study the various parameters affecting the non-solvent nanoprecipitation method by investigating the colloidal properties of BSA nanoparticles. The effect of several parameters (such as protein concentration, solvent/non-solvent volume ratio, non-solvent injection speed, ionic strength, crosslinker, and process variation) on the size, charge density or electrokinetic properties, shape, and morphology of nanoparticles was investigated.

To the best of our knowledge, this is the first work that investigates the factors affecting the non-solvent nanoprecipitation method and their impact on the properties of nanoparticles in the absence of some toxic compounds usually used in drug formulations.

## 2. Materials and Methods

### 2.1. Chemical Reagents

Bovine serum albumin (BSA) and sodium hydroxide were obtained from Sigma–Aldrich (MO, USA). Glutaraldehyde (crosslinker) solution (25%) and Ethanol 96% were provided by VWR BDH Prolabo (VWR, Paris, France). DPBS (+) and dimethyl sulfoxide (DMSO) were purchased from Sigma-Aldrich. Disodium phosphate and monosodium phosphate were provided by Merck GmbH. Electrophoresis gel components, which consisted of acrylamide 40%, Tris electrophoresis purity reagent, sodium dodecyl sulfate, ammonium persulfate, and tetramethylethylenediamine (TEMED), were all provided by Biorad. Ultra-pure water was purified using a synergy unit system (Millipore, Villeurbanne, France).

### 2.2. Preparation of BSA Nanoparticles

BSA nanoparticles were prepared by nanoprecipitation method using different approaches that vary in the manner of addition of the non-solvent (ethanol). Ethanol was added either by direct addition, using a syringe pump, or by using a burette with free flow, depending on the tested parameters.

#### 2.2.1. Effect of BSA Concentration and Solvent/Non-Solvent Volume Ratio

**Figure 2:**
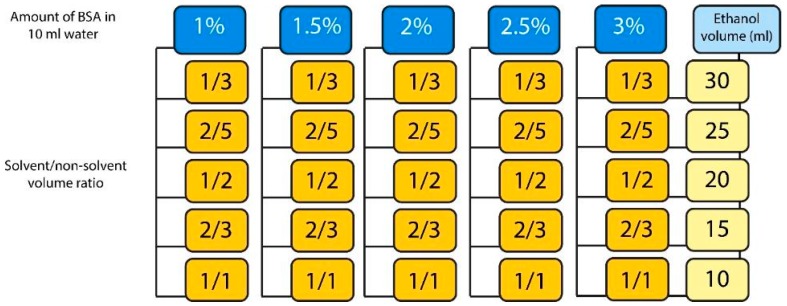
Amount of BSA and solvent/non-solvent volume ratios used to prepare BSA nanoparticles.

Five BSA aqueous solutions (50 mL) at concentrations of 1%, 1.5%, 2%, 2.5%, and 3% *w*/*v* were prepared. Each solution was divided into five different volumes of 10 mL for each. BSA was precipitated in these solutions by direct addition of ethanol (a non-solvent phase) at different solvent/non-solvent volume ratios (S/NS) equal to 1/3, 2/5, 1/2, 2/3, and 1/1. The S/NS and ethanol volumes chosen are listed and presented in Figure 2.

#### 2.2.2. Effect of Ethanol Injection Rate

Two BSA solutions of 1% (*w*/*v*) concentration were prepared. The first solution was prepared by dissolving BSA in water under constant stirring speed (500 rpm) at room temperature and at pH 7 adjusted by adding 0.1 N NaOH (BSA reduces the pH of water to around 6.5). The other one was prepared by dissolving BSA in a phosphate buffer (10 mM, pH 7) solution under the same stirring and temperature conditions. Then, 5 mL of each solution were taken consecutively and 15 mL of ethanol (at S/NS volume ratio of 1/3) were added using a syringe pump at different injection rates (0.1, 0.25, 0.5, 1, and 2 mL/min) under the same stirring and temperature conditions.

#### 2.2.3. Effect of Ionic Strength

**Figure 3:**
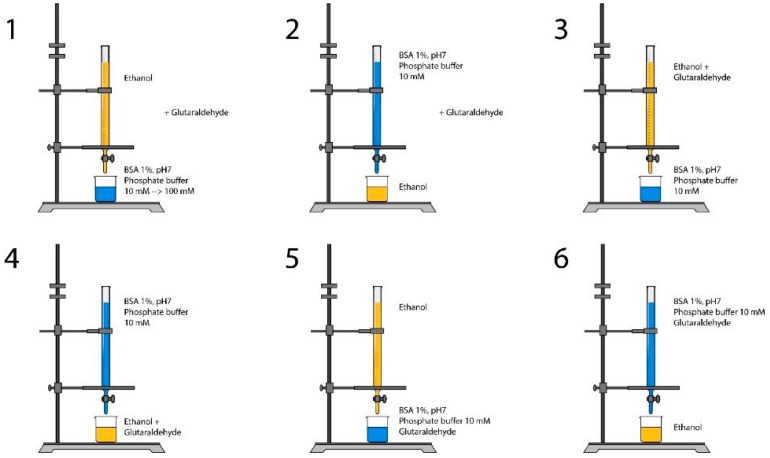
Different nanoprecipitation processes to prepare BSA nanoparticles. In 1 & 2, glutaraldehyde was added after the mixing of solvent and non-solvent together.

BSA solutions 1% (*w*/*v*) were prepared in phosphate buffer solutions with different ionic strengths (10, 20, 30, 50, and 100 mM) at pH 7 under magnetic stirring (500 rpm). Then, 15 mL of ethanol solution were added using a burette with free flow to 5 mL of each of the prepared 1% BSA solutions. After desolvation, 20 μL of 8% (*v*/*v*) glutaraldehyde crosslinker were added (Figure 3(1)).

#### 2.2.4. Effect of pH

BSA 1% (*w*/*v*) aqueous solution was prepared. This solution was divided into two equal volumes of 25 mL each. After that, each of these two volumes were divided into five subvolumes of 5 mL each. These subvolumes were precipitated by adding 15 mL ethanol under stirring (S/NS = 1/3). 20 μL of glutaraldehyde 8% were then added to five of these subvolumes directly after the addition of ethanol. Ethanol was then evaporated at 30 °C and 100 rpm and pH was adjusted to each sample (crosslinked and non-crosslinked) at 3, 5, 7, 9, and 11 using HCl and NaOH.

Another BSA 1% (*w*/*v*) solution was prepared in 1 mM NaCl. Ethanol was added under magnetic stirring at S/NS volume ratio of 1/3. After precipitation, ethanol was evaporated at 30 °C and 100 rpm. The obtained solution was divided into five equal volumes and pH was adjusted to each volume at 3, 5, 7, 9, and 11.

#### 2.2.5. Effect of Process Variation

In addition, different modifications in the method were examined as shown in Figure 2. BSA 1% (*w*/*v*) solutions in phosphate buffer, pH7, 10 mM were mixed with ethanol (S/NS volume ratio 1/3) using a burette with free flow and using different installations under magnetic stirring (500 rpm). Glutaraldehyde was added either with ethanol, or with BSA solution, or after the precipitation, according to Figure 3.

### 2.3. Determination of Nanoparticle Yield

1 mL of nanoparticles prepared from BSA 1% sample was centrifuged at 6000 g for 5 min. After centrifugation, supernatant was collected. Absorbance of the supernatant was measured and the amount of BSA was determined using standard Bradford protein assay. After quantification of free BSA in the supernatant, the yield of nanoparticles formation could be calculated with the following equation

$$Yield=\frac{{\left[BSA\right]}_{total}-{\left[BSA\right]}_{free}}{{\left[BSA\right]}_{total}}\times 100$$

where [*BSA*]*_total_* is the total concentration added and [*BSA*]*_free_* is the concentration of dissolved *BSA* remaining in the supernatant after centrifugation.

### 2.4. Stability of BSA Nanoparticles

Nanoparticles from BSA 1% (*w*/*v*) samples prepared before and adjusted at pH 3, 5, 7, 9, and 11 were submitted to a UV scan at 280 nm for 5 min. Absorbance was plotted against time for both crosslinked and non-crosslinked particles.

In addition, BSA nanoparticles and free BSA originated from BSA 1% aqueous solution (*w*/*v*) were subjected to polyacrylamide gel electrophoresis. 20 µL of BSA nanoparticles and dissolved BSA samples with a concentration of 0.31 µg/µL were introduced in the gel at room temperature in the presence of an electric current of 10 mA.

### 2.5. Characterization of BSA Nanoparticles

All prepared BSA nanoparticle dispersions were characterized in terms of size, electrokinetic properties, and morphology. Size and zeta potential were measured using a Malvern Zetasizer (model nano ZS) by photon correlation spectroscopy and electrophoretic mobility measurement respectively. Size and coefficient of variation were calculated by means of the Strokes–Einstein equation from the diffusion coefficient measured by dynamic light scattering (DLS) [35]. Zeta potential was calculated from the measured electrophoretic mobility using the Helmholtz–Smoluchowski equation [36]. Nanoparticles morphology was examined by scanning electron microscopy (SEM) and transmission electron microscopy (TEM). SEM was performed using a FEI Quanta 250 FEG microscope at the “Centre Technologique des Microstructures” (CTμ) at the University of Lyon (Villeurbanne, France). A drop of diluted aqueous suspension of nanoparticles was deposited on a flat steel holder and dried at room temperature. The sample was finally coated under vacuum by cathodic sputtering with copper. The samples were observed by SEM under an accelerating voltage of 15 kV.

TEM was performed with a Philips CM120 microscope at the “Centre Technologique des Microstructures” (CTμ) at the University of Lyon (Villeurbanne, France). 10 µL of suspension was deposited on a microscope grid (copper support covered with carbon) and slowly dried in open air. The dry samples were observed by TEM under 120 kV acceleration voltage. Absorbance was measured by Cary UV-visible spectrophotometer (Varian, Australia) and values were given according to the Beer–Lambert equation at wavelength of 280 nm.

### 2.6. Statistical Analysis

To assess the significant differences between values, statistical analysis was carried out using the student’s *t*-test. A *p* value < 0.05 was accepted as the level of significance.

## 3. Results and Discussions

### 3.1. Effect of BSA Concentration and Solvent/Non-Solvent Volume Ratio

**Figure 4:**
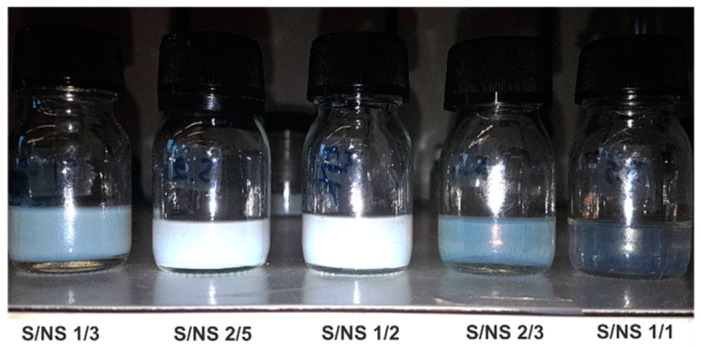
The variation in color of 1% BSA formulations at different S/NS volume ratios.

BSA nanoparticles were prepared using various concentrations and different volumes of ethanol (Figure 2). From visual inspection of samples, it was obvious that at different S/NS ratios, there was a difference in the properties of nanoparticles. Samples prepared at S/NS = 1/3 (30 mL ethanol) had a fading transparent white color. By increasing the S/NS ratio, the color (i.e., opacity) increased until it lost its transparency at S/NS = 1/2 (where the volume of ethanol was twice the volume of water). The successive increase in S/NS ratios (at 2/3 and 1/1) led to a complete disappearance (Figure 4). This alternation in turbidity can be attributed to the particle number, particle size, and size distribution.

**Figure 5:**
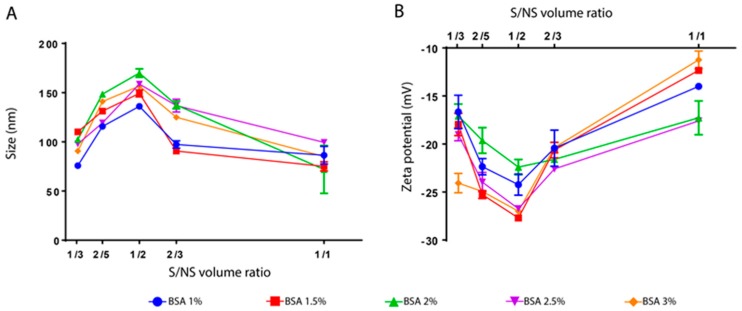
The size (**A**) and zeta potential (**B**) of BSA nanoparticles as a function of S/NS volume ratio at different BSA percentages (from 1 to 3%). Measurements were performed at room temperature with pH between 6.8 and 7. Measurements from separate assays were combined (*n* = 6) and represented as mean ± SD.

For further investigation, the variation of both particle size and zeta potential was studied as a function of S/NS ratio for all the BSA concentrations used in this study. Figure 5A shows the relation between nanoparticle size and S/NS for different BSA concentrations after the evaporation of ethanol. For all BSA concentrations, the size of nanoparticles increased by 50 nm with an increase in the S/NS ratio from 1/3 to 1/2. The successive increase in S/NS ratio (i.e., >1/2) led to a significant reduction in size to reach its minimum for all BSA concentration at S/NS ratio of 1/1 (Figure 5A). This alternating behavior in size could explain the color fading observed in Figure 4.

The concept of nanoprecipitation is based on the interfacial turbulence generated during solvent displacement. As a consequence, a violent spreading of the added non-solvent will occur thanks to the mutual miscibility of the two solvents. During the diffusion, the protein will exceed its thermodynamic solubility limit and the system will behave in such a way to induce the nucleation of protein nanoparticles [37,38].

**Figure 6:**
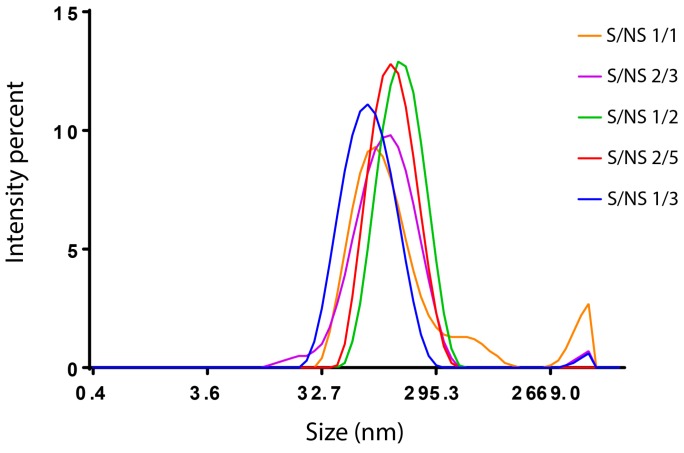
BSA particle size distribution obtained by Malvern Zetasizer. BSA 2%, at room temperature, and pH between 6.8 and 7. At different solvent/non-solvent volume ratios: S/NS 1/3; S/NS 2/5; S/NS 1/2; S/NS 2/3; S/NS 1/1.

In other words, according to this theory, the formation of nanoparticles depends on the volume of non-solvent added. If the volume of the non-solvent is not enough for the solute to reach its supersaturation, precipitation will not occur or it will lead to malformed particles with a wide size distribution. This could explain the interruption in size by using a solvent/non-solvent volume ratio >1/2. Simply, the amount of ethanol was not enough to achieve proper precipitation. The size distribution of the nanoparticles obtained by dynamic light scattering (Malvern Zetasizer, Malvern, France) is shown in Figure 6. It was found that for S/NS volume ratios 1/3, 2/5, and 1/2, BSA nanoparticles with unimodal size distribution were observed and became narrower by increasing the S/NS ratio from 1/3 to 1/2. However, a small amount of aggregation is observed in the sample of S/NS 1/3 (Figure 6). In addition, when the S/NS volume ratio was 2/3, the peak lost its perfect Gaussian shape and shifted towards the left, indicating an increase in polydispersity. A small peak was also observed, indicating the presence of aggregation (Figure 6). Finally, at an S/NS volume ratio of 1/1, an irregular peak appeared, indicating the high polydispersity of the particles with a large signal of aggregation (Figure 6).

These results indicate that when the S/NS is above 1/2, the size distribution will be very high and the measurement will not be accurate. These results are in correlation with what Stainmesse et al. suggested. While preparing poly-ε-caprolactone nanoparticles by nanoprecipitation, they observed that when the solvent/non-solvent volume ratio is equal to 1 and above, the particle size cannot be determined [39].

In addition, the yield of nanoparticle formation of samples of BSA 1% and S/NS 1/3 was calculated. It was found that the yield of nanoparticle formation for these parameters was approximately 70%. In another study, BSA nanoparticles prepared by the coacervation process with the use of a crosslinker show a yield of 95%, indicating the importance of the experimental conditions for reproducibility and reliability [30].

The effect of the S/NS volume ratio on the zeta potential was also studied (Figure 5B). A similar alternating behavior in zeta potential was also observed, as in the case of particle size. It was found that the zeta potential value decreased by increasing the S/NS ratio from 1/3, 2/5, to 1/2, then it increased to achieve its maximum at S/NS of 1/1. The same previous explanation could be adopted in this case; samples could be highly polydisperse so the measurement is not accurate at high S/NS volume ratios. In addition, it is known that the high absolute zeta potential value refers to the high colloidal stability of nanoparticles [40]. As clearly seen from Figure 5B, BSA nanoparticles possessed the highest colloidal stability at an S/NS volume ratio of 1/2.

Furthermore, the effect of BSA concentration on the size and zeta potential of nanoparticles was evaluated. Only a slight difference in size exists by increasing BSA concentration. This result is in agreement with the work of Langer et al., 2003, where they prepared human serum albumin (HSA) nanoparticles by nanoprecipitation method [41]. They found that HSA concentration between 25 and 100 mg/mL has only a slight influence on the particle size (between 150 and 170 nm). However, in another study, the increase of BSA concentration from 12 to 100 mg/mL led to an increase of particle size from 75 to 135 nm [30]. Oppositely, Rahimnejad et al. showed that increasing BSA concentration from 5 to 30 mg/mL led to the reduction of particle size from 204 to 145.7 nm [9].

This contradiction in the literature can be explained by the presence of different parameters that affect nanoparticle size for each study. Besides protein concentration, it is known that non-solvent type, non-solvent volume, the presence of a drug, and a drug concentration could affect the process of precipitation by making changes in the environment or by altering conformation of the protein. Consequently, this leads to a variation in the patterns of how each parameter affects the colloidal properties of nanoparticles [42].

As for zeta potential, BSA concentration does not seem to affect its value (Figure 5B). Particles from all formulations have zeta potential values between −15 and −25 mV except for the sample that was prepared at S/NS 1/1 volume ratio (−10 mV) (Figure 5B). These values could indicate the good colloidal stability of particles since the increase of zeta potential value leads to less attraction between particles and consequently less aggregation [40].

**Figure 7:**
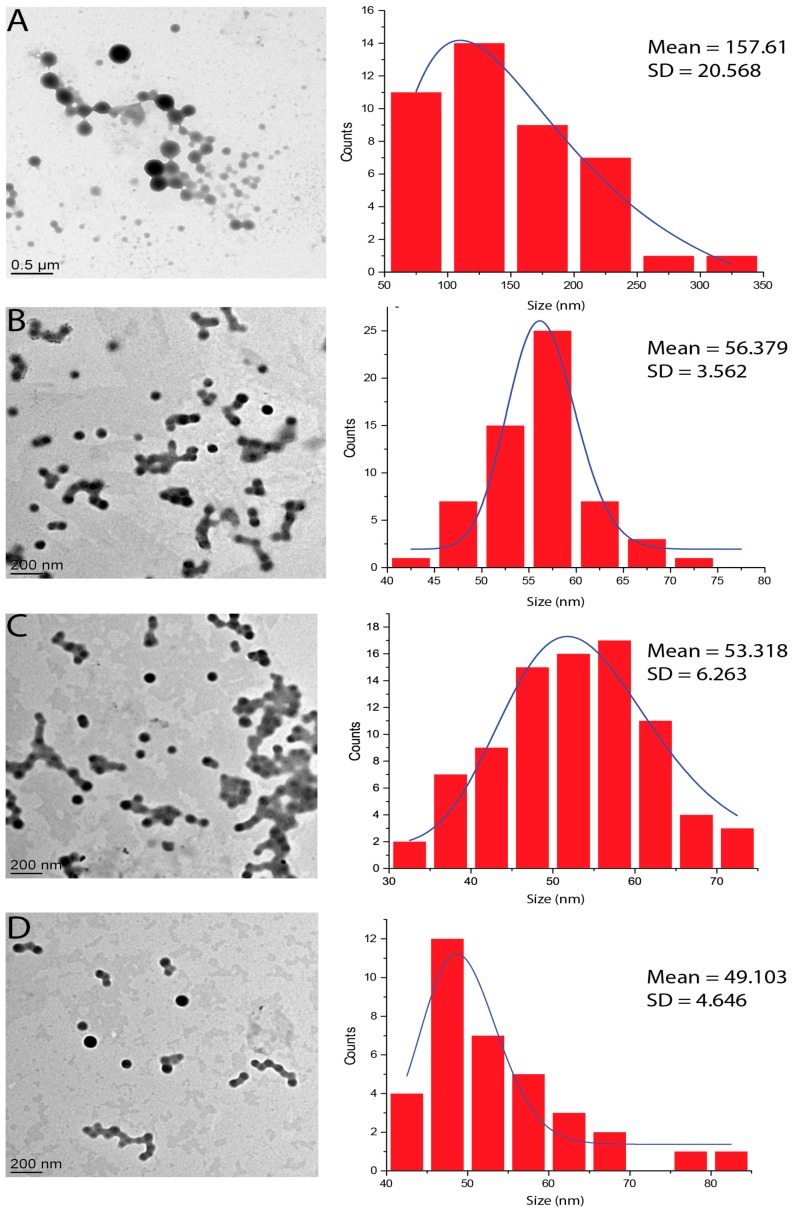
TEM images for different samples and their associated particle size histogram: (**A**) BSA2.5%, S/NS2/5; (**B**) BSA2.5%, S/NS1/2; (**C**) BSA2%, S/NS2/5; and (**D**) BSA2%, S/NS1/2. Images were analyzed using ImageJ and results are represented as mean size and standard deviation (SD).

The morphology of BSA nanoparticles was examined by transmission electron microscopy (TEM) (Figure 7). TEM images show BSA spherical-shaped nanoparticles. However, the size of nanoparticles obtained by the statistical analysis of TEM images (Figure 7) is lower than the size obtained by DLS for the same samples (Figure 6A) [43]. This difference in size is expected since TEM allows the observation of samples in a dry state in which particles are in the most compact form. In addition, the yield of nanoparticle formation of BSA 2% samples oscillated from 68% and 70%.

### 3.2. Effect of Ethanol Injection Rate

**Figure 8:**
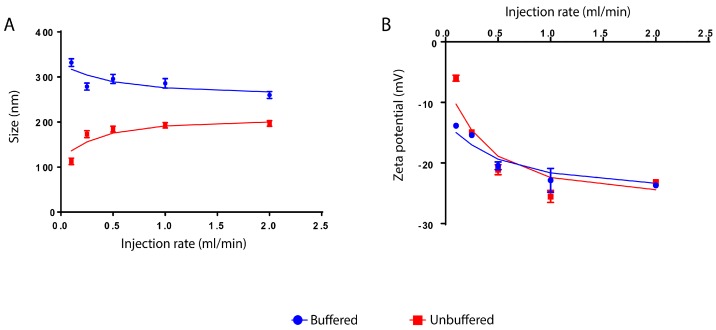
The effect of ethanol injection rate on the size (**A**) and zeta potential values (**B**) of BSA nanoparticles dispersed in water and in phosphate buffer 10 mM, at pH 7. Measurements from separate assays were combined (*n* = 6) and showed in mean ± SD.

At constant pH 7, the effect of the ethanol addition rate on nanoparticle size was investigated for BSA aqueous solution and for BSA prepared in phosphate buffer solution (PBS, Arlington, VA, USA, 10 mM). In the case of BSA prepared in PBS buffer solution, the BSA particle size was decreased by increasing the flow rate from 0.1 to 0.25 mL/min and then became almost constant from 0.25 to 2 mL/min. In the case of normal BSA aqueous solution, the particle size was considerably increased by increasing the ethanol addition rate below 0.5 mL/min. However, the further increase in ethanol addition rate (>0.5 mL/min) has no substantial effect and the size became almost unchanged (Figure 8A).

The reduction in size in the buffered samples can be explained by the classic theory of nucleation. As it is well known, the non-solvents’ precipitation process is divided into four phases, including generation of supersaturation, nucleation, growth, and coagulation. It is also known that the supersaturation conditions could affect the size of final nanoparticles. The high and rapid supersaturation will lead to an increase in the nucleation rate, which consequently induces the formation of a larger number of nuclei in the initial stage of the nucleation process, giving rise to fast particle growth and the formation of smaller nanoparticles [17,18]. In addition, the degree of supersaturation is governed by the addition rate of ethanol. Therefore, when the ethanol injection rate increases, the supersaturation rate and degree of nucleation increase and finally smaller nanoparticles can be obtained [17]. However, this effect is not significant above an ethanol addition rate of 0.5 mL/min.

González et al. [30] used BSA buffered solution to prepare nanoparticles by precipitation. They showed that by increasing the ethanol flow rate from 1 to 10 mL/min, the size of nanoparticles decreased from 170 to 90 nm [30]. Moreover, Kakran et al. prepared curcumin nanoparticles using an ethanol solution of curcumin and water as the non-solvent. They also noticed that the nanoparticle size decreased by increasing the flow rate of the non-solvent [44].

However, the increase of particle size, in the case of unbuffered samples, cannot be explained by this theory. In fact, the increase in size by increasing the ethanol addition speed contradicts it. For instance, Langer et al. have prepared HSA nanoparticles by precipitation of HSA units from HSA aqueous solution using ethanol as the non-solvent. It was noticed that the rate of ethanol addition did not affect nanoparticle size. The size of nanoparticles (180 nm) remained constant despite the change of ethanol addition rate from 0.5 to 2 mL/min [41], which perfectly fits with the results obtained in Figure 8A.

In addition, it is clear that the size of BSA nanoparticles prepared in PBS buffer medium is higher than those prepared in normal aqueous medium (Figure 8A). This phenomenon can be attributed to the buffer solution in which the buffer ions cause the reduction of surface charge density of the particles. This in turn reduces the electrostatic repulsion and leads to attraction and fusion between particles and consequently an increase in particle size [45].

**Figure 9:**
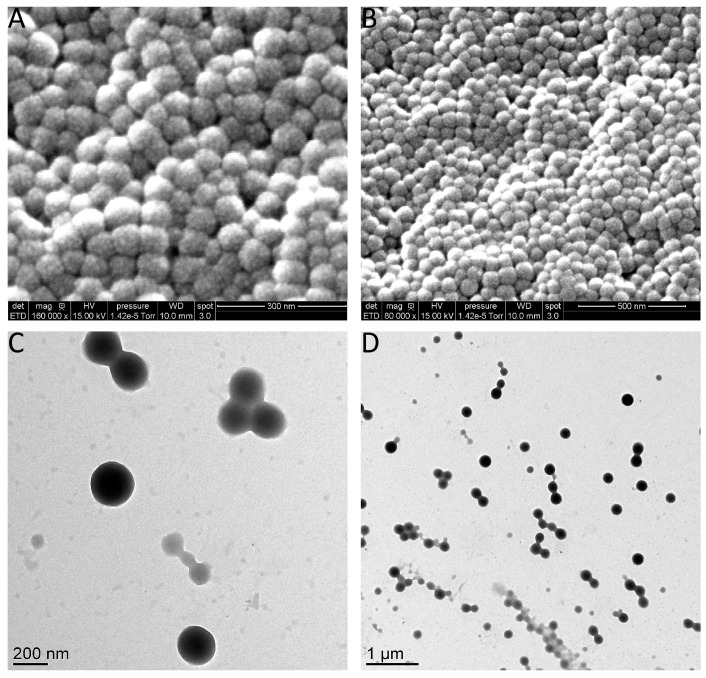
Scanning electron microscopy images of BSA nanoparticles prepared in 10 mM phosphate buffer with (**A**) 2 mL/min injection rate; (**B**) 0.25 mL/min injection rate. Transmission electron microscopy of (**C**) 2 mL/min injection rate; and (**D**) 0.25 mL/min injection rate.

As for zeta potential, by increasing the rate of ethanol injection, it was observed that the values decreased from −13 mV to −23 mV for the buffered medium and from −5 to −23 mV for the unbuffered medium. However, in both cases, zeta potential values above 0.25 mL/min injection rate were almost the same (Figure 8B). Moreover, SEM and TEM images of the buffered samples prepared at the ethanol injection rate of 0.25 mL/min and 2 mL/min are shown in Figure 9.

### 3.3. Effect of Ionic Strength

The effect of ionic strength was done according to Figure 3(1). Ionic strength markedly affects the coagulation of BSA molecules. By changing the ionic strength of PBS from 100 mM to 20 mM, it was observed that the coagulation led to the precipitation of coarse particles while the use of PBS at 10 mM provided a successful nanoprecipitation. At pH 7 and 10 mM buffer concentration, BSA molecules possess a net negative charge. This leads to electrostatic repulsion between protein molecules that slow down the aggregation of molecules, allowing the formation of nano-sized particles (185 nm). At higher PBS concentration (above 10 mM), the salt is responsible for shielding the surface charge of protein molecules; positive ions attract to the negatively charged BSA molecules and consequently reduce the net charge of BSA molecules. Thus, electrostatic repulsion between protein molecules is weakened and amorphous aggregates are readily formed via non-specific interaction (mainly of hydrophobic nature). In fact, the non-polar amino acid of BSA molecules tends to bind with a hydrophobic part of its surface. Hydrophobic interactions lead to coagulation resulting in size increase and sedimentation.

### 3.4. Effect of pH

**Figure 10:**
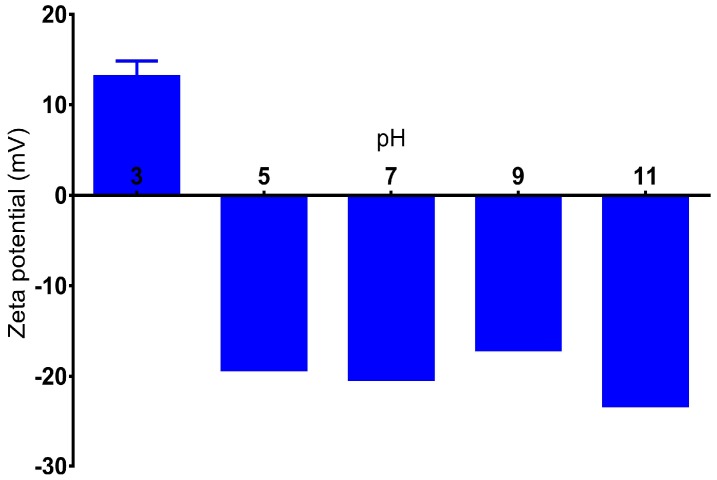
The influence of pH on the zeta potential of BSA nanoparticles prepared in 1 mM NaCl. Measurements from separate assays were combined (*n* = 6) and showed in mean ± SD.

The change in the pH value shows a dramatic effect on the zeta potential of BSA nanoparticles. Particles prepared in 1 mM NaCl solution show a positive zeta potential value (15 mV) when pH was adjusted to 3. At higher pH, particles drop to the negative values between −18 and −23 mV for the pH range of 5 to 11 (Figure 10). This result is in correlation with the work of Langer at al [41] where they studied the effect of pH on the zeta potential of HSA particles, and the same behavior was observed. This variation of surface charge could be explained by the characteristic of the protein to change its conformation at different pH values. In addition, the pH value could affect the charge of the protein, leading to different profiles of particle formation and characteristics [41].

**Figure 11:**
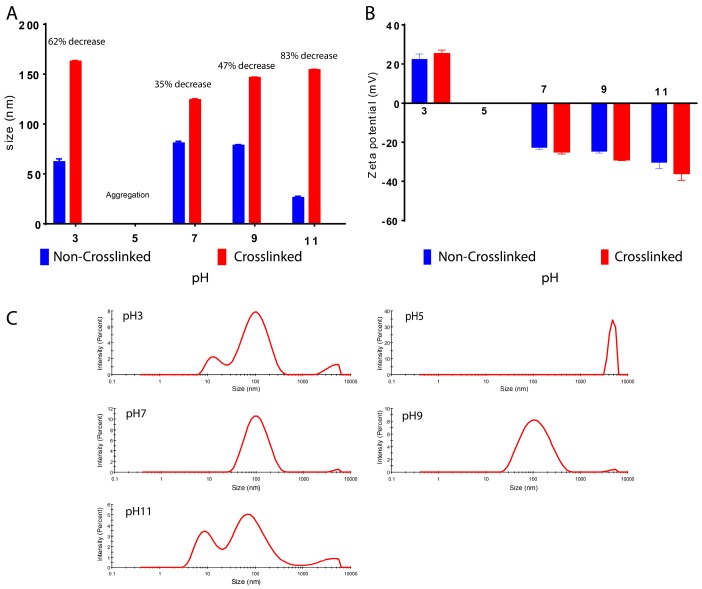
(**A**) Size of crosslinked and non-crosslinked BSA nanoparticles; (**B**) zeta potential; and (**C**) size distribution of non-crosslinked BSA nanoparticles at different pH values.

Furthermore, dissociation pH of BSA nanoparticles was investigated. Crosslinked and non-crosslinked particles were prepared and dispersed in water. The pH was adjusted after the precipitation and the size of crosslinked particles were compared to the non-crosslinked ones (Figure 11A). It was found that the size of non-crosslinked particles was significantly lower than that of the crosslinked particles. For the crosslinked particles, the size varied from 162 to 124 nm, depending on the pH values. However, the non-crosslinked particle size ranged from 81 to 26 nm. This could probably indicate a dissociation phenomenon [41]. By changing the pH values, the difference between crosslinked and non-crosslinked particles became bigger. This may indicate a dissociation or aggregation of the non-crosslinked particles. At pH 11, the non-crosslinked particles had lost 83% of their size compared to the crosslinked ones. At pH 3, 62% of the particle size was lost. By viewing the size distribution of these two samples, it can be observed that several peaks appeared, including a peak at approximately 10 nm for both cases, indicating the presence of a smaller population of BSA nanoparticles that can be caused by dissociation. In addition, at pH 7 and 9, the decrease in size was relatively lower than the previous samples (35% and 47%, respectively). As for zeta potential, crosslinked particles have higher absolute values compared to the non-crosslinked particles for all pH points measured. It was also shown that the zeta potential decreased by increasing pH value from 20 mV at pH 3 to approximately −20 mV at pH 7 and straight to −30 mV at pH 11 (Figure 11B). However, the size distribution graphs of these two samples show a single major peak, indicating the presence of one population of nanoparticles, and one small peak higher than 1000 nm as a sign of a small amount of aggregation, probably caused by the lost BSA units (Figure 11C). Moreover, at pH 5, a rapid aggregation was observed for both crosslinked and non-crosslinked samples. This is normal due to the near isoelectric point of BSA (pI 4.7) where the electric repulsion is nullified and the particles are attracted to each other causing aggregation (Figure 11C).

### 3.5. Stability Investigation

**Figure 12:**
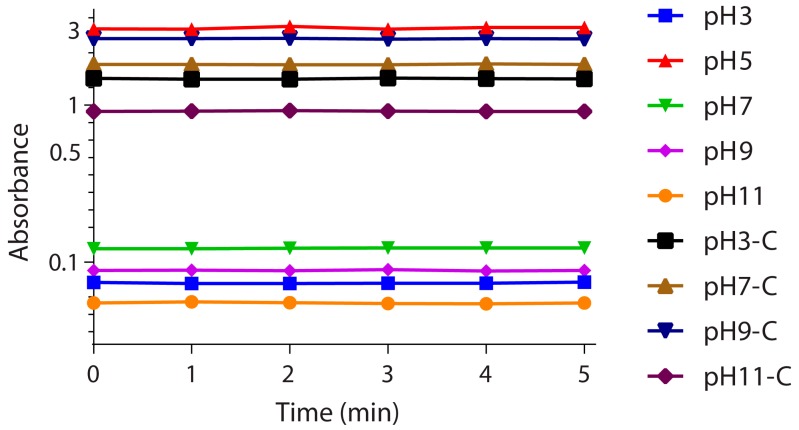
Absorbance of non-crosslinked and crosslinked (C) BSA nanoparticles at different pH in function of time at a constant wavelength 280 nm.

The stability of BSA nanoparticles was investigated by measuring the absorbance of particles for five minutes at 280 nm wavelength at different pH values (3, 5, 7, 9, and 11) (Figure 12). It was found that at pH 5, the absorbance value was relatively high (approximately 3) compared to other pH values. This could be explained by the formation of aggregations in the medium at this pH. Moreover, at pH 3 and 11, the absorbance was lower than the other values (0.07 and 0.05, respectively). This could be due to the lower-sized particles observed in Figure 11 for these two pH values. Furthermore, the absorbance spectra of all pH values were constant, indicating a stability of the formulations.

**Figure 13:**
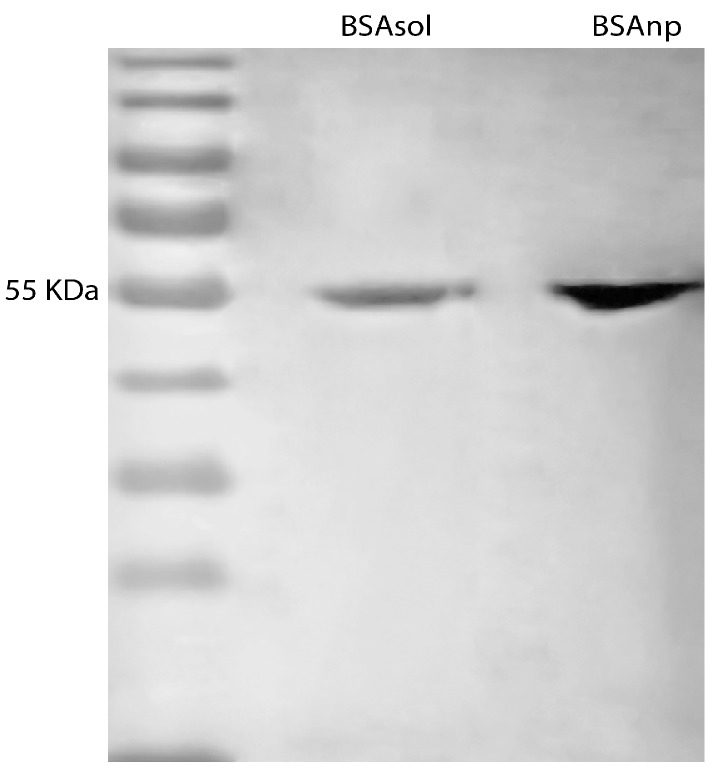
Acrylamid gel electrophoresis for free BSA and BSA nanoparticles of 0.3 mg/mL each.

In addition, BSA nanoparticles were migrated in an acrylamide gel in order to investigate the signs of decomposition (Figure 13). It was found that Free BSA stain and nanoparticle BSA stain have the same migration rate at 55 kDa. Gel electrophoresis is based on the separation of species in the sample according to their size, so the presence of a single stain indicates the presence of uniformly-sized species [46]. In the case of BSA nanoparticles, the presence of a single stain indicates that the nanoparticles are not decomposing after preparation. Otherwise, stains of different sizes should be observed in case of particle aggregation or decomposition.

### 3.6. Effect of Method Variation

**Figure 14:**
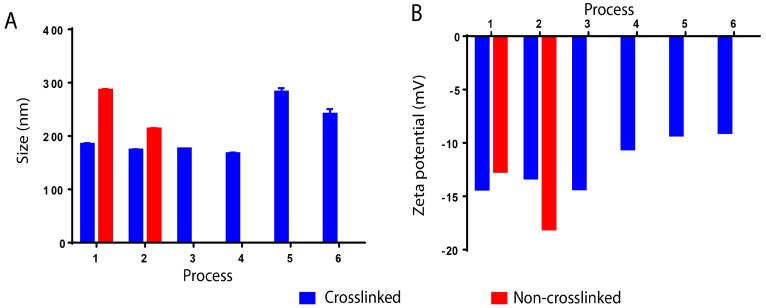
The effect of process variation on the size and zeta potential values of BSA nanoparticles. Measurements from separate assays were combined (*n* = 6) and expressed as mean ± SD.

Different variations of the method were tested as shown in Figure 3 and the effect of these variations on the size and zeta potential values was investigated. Figure 14 shows that these variations had no effect on the size of nanoparticles, which stayed around 180 nm. However, in the processes 5 and 6, where glutaraldehyde was added to the solvent, the size increased to approximately 250 nm (Figure 14). The same goes for a zeta potential that stayed constant from processes 1 to 4 (about −13 mV), then decreased slightly in processes 5 and 6 where glutaraldehyde was added directly to the solvent phase for about −9 mV. In addition, for processes 1 and 2, samples were taken before crosslinking of nanoparticles. It was found that in the absence of the crosslinker, the size of nanoparticles turned to be high in both cases, 284 nm for process 1 and 214 nm for process 2. On the other hand, zeta potential values decreased in process 1 to −12.6 mV and increased for process 2 to −18 mV (Figure 14).

In this work, it was confirmed that the experimental parameters of the nanoprecipitation method could dramatically affect the colloidal properties of the nanoparticles. In addition, some experimental parameter changes can lead to aggregation or polydispersity in the samples. The values of size, zeta potential, and polydispersity index obtained by Zetasizer can reflect the effect of these parameters and are summarized in Table 2.

## 4. Conclusions

In this work, the effect of experimental parameters on the colloidal properties of BSA nanoparticles prepared by nanoprecipitation method was studied. BSA nanoparticles were produced without the use of glutaraldehyde as a crosslinker or any other toxic compound with various colloidal properties depending on the variation of the experimental parameters. In addition, parameters such as solvent/non-solvent volume ratio, pH, and non-solvent addition speed can dramatically affect the size and zeta potential of the particles. On the other hand, some parameters can lead to aggregation or dissociation of the particles due to instability. Therefore, this work was able to identify the optimal preparation conditions of BSA nanoparticles by nanoprecipitation method that are suitable for in vivo drug delivery since no toxic compounds were used in the formulation.

## Figures and Tables

**Table 1:** Active compounds encapsulated within bovine serum albumin (BSA) nanoparticles.

Active Compound	Preparation Method	Goal	Ref.
5-Fluorouracil	Coacervation	In vitro drug release profile	[8]
10-hydroxycamptothecin	W/O Emulsion	Characterization of nanoparticles and in vitro targeted delivery profile	[19]
Aspirin	Coacervation	Preparing formulations for intra-articular therapy	[20]
Cefamandole nafate (antibiotic)	W/O emulsion	Prevention of medical device-related infections	[21]
Doxorubicin	Desolvation	Tumor inhibition of murine ascites hepatoma	[22]
γ-Interferon	Coacervation	Targeting of γ-interferon into macrophages	[23]
Ganciclovir	Coacervstion	In vitro drug release profile of nanoparticles	[24]
Methotrexate	Coacervation	Study the biodistribution of nanoparticles	[25]
Oligonucleotides	Coacervation	Delivery of antisense oligonucleotides	[26]
Paclitaxel	Deslovation	Characterization and in vitro targeted delivery profile	[27]
Pilocarpine	Desolvation	The effect of bioadhesive polymer on the in vivo activity of nanoparticles	[28]
Sodium Ferulate	Desolvation	Liver targeting	[29]

**Table 2:** Size, zeta potential, and polydispersity index of BSA nanoparticles prepared by nanoprecipitation method and the effect of the various experimental parameters used in this work.

Parameters		Mean Size (nm)	Mean Zeta Potential (mV)	PDI
BSA % at S/NS 1/2	1%	137	−24.1	0.228
	1.50%	148	−27.7	0.217
	2%	165	−23.5	0.116
	2.50%	158	−26.6	0.152
	3%	150	−27.2	0.144
S/NS at BSA 2%	1/3	101	−17	0.25
	2/5	148	−19.7	0.289
	1/2	165	−23.5	0.116
	2/3	140	−22.9	0.372
	1/1	77	−18.2	0.818
Ethanol addtion speed (ml/min) Unbuffered	0.10	196	−23.1	0.137
	0.25	192	−25.5	0.165
	0.50	184	−21.1	0.221
	1.00	173	−14.9	0.291
	2.00	112	−6	0.243
pH	3	64	22.5	0.449
	5	Aggregation	Aggregation	
	7	82	−21.9	0.29
	9	79	−24.3	0.45
	11	26	−27.6	0.803
[PB] mM	10	184.5667	−14.3	0.055
	20	Aggregation	Aggregation	
	30	Aggregation	Aggregation	
	50	Aggregation	Aggregation	
	100	Aggregation	Aggregation	
Process number	1	184	−14.3	0.055
	2	173	−13.3	0.142
	3	175	−14.3	0.095
	4	167	−10.56	0.129
	5	282	−9.2	0.265
	6	240	−9	0.192
